# Women in Epidemiology

**DOI:** 10.1017/ash.2023.151

**Published:** 2023-04-17

**Authors:** Diana Vilar-Compte

**Affiliations:** Department of Infectious Diseases, Instituto Nacional de Cancerologia, Mexico City, Tlalpan, Mexico

## Tell us about your career journey and what it is like to be a female-identifying epidemiologist practicing in your country

I come from a physician family; I am 1 of 5 physicians but the first woman. I was expected to be an otolaryngologist like my great-grandfather and grandfather in Barcelona and my father in Mexico. My grandfather and father have been important role models, exemplifying what it means to be a doctor and scientist.

Early in my career, I was interested in academia, internal medicine, pathophysiology, and microbiology, and by the end of medical school, I liked infectious diseases, rheumatology, public health, and epidemiology. I wanted to be a clinical researcher, and during a compulsory year of social service, I was exposed to epidemiology by Dr. Juan Calva, an epidemiologist and infectious diseases physician who was one of the first clinical epidemiologists in Mexico. Simultaneously, I met Dr. Samuel Ponce de León, the first hospital epidemiologist in the country. This was likely the beginning of my hospital epidemiology and infection control career.

Later, in the 1990s, I decided to pursue a master’s degree in epidemiology under the supervision of Dr. Patricia Volkow at Instituto Nacional de Cancerologia, a cancer hospital in Mexico City. Dr. Volkow had started an infection control program and an intravascular catheter team. She taught me the importance of standardized procedures, high-quality medical supplies, and systematic data gathering. She suggested studying surgical infections, publishing the data, and changing policy. It seemed exciting, but at the time, I didn’t realize the challenges I would face as a young women epidemiologist in an environment full of male physicians, often unwilling to change or question long-standing practices. However, my convictions and support from the head of the infectious diseases department and a new hospital director helped create necessary change. Thirty years later, I am grateful for the opportunity to make surgical oncology safer through research and policy improvements.

## What are the unique challenges for women in medicine in your country?

In Mexico, like other countries, more women than men are enrolled in medical school. However, many inequities still need to be addressed, with very few women as section chiefs and in directorate positions. Only some female physicians in leadership positions can enact policy changes to reduce the hurdles other female physicians face.

Moreover, Mexico is a patriarchal society with high rates of workplace hostility, discrimination, and harassment despite government protections. A study from one of the largest academic hospitals in Mexico City found that 70% of women physicians felt the need to restrain their opinions in the academic setting to avoid uncomfortable comments from male colleagues, 93% had experienced discomfort at least once due to comments from male colleagues, and 57% had been harassed.

In Mexico, women’s daily challenges are deeply rooted in cultural norms. Patients still commonly refer to women physicians as “señorita” instead of “doctora.” This practice, while slowly changing, reflects a culture in which women are expected to play other roles. Therefore, women in medicine must fight harder to be respected and recognized. In addition, female healthcare workers in Mexico are also at risk of gender violence and other crimes experienced while commuting to and from work. Young women physicians working in remote areas face the highest risk of workplace violence and harassment. These circumstances adversely impact female physicians and the patient–physician relationship.^1–4^

Other challenges I’ve faced are likely shared by anyone practicing medicine in a low- or middle-income country. Infection control programs are chronically underresourced and understaffed, and we were almost invisible until the COVID-19 pandemic. We have few human or technical resources and even fewer resources for research. Moreover, we struggle for the visibility of our research by colleagues abroad.

## What were some barriers in your career and how did you address them?

Early in residency, I became ill with a physical disability that kept me out of medicine for almost a year and forced me to make drastic career choices. Although it was a difficult time, I learned to dare to be different, persevere, and commit more deeply to my career goals.

In hindsight, my illness accelerated the type of career I aspired to have, and becoming a patient undoubtedly made me a better doctor. In the end, I exceeded all expectations for myself, but not without setbacks. I learned that the medical profession could sometimes be hostile to those who must adapt to challenging personal circumstances.

As a young professional in infection control and hospital epidemiology, I faced challenges convincing other physicians and administrators to make evidence-based practice changes to make healthcare safer. I learned with time that modulated and precise actions, prioritized by importance, were keys to success. In addition, independence in my research and expanded professional networks helped me to overcome these challenges. An important lesson learned is that you cannot win all battles or prevent all hospital-acquired infections. Learned strategies enabled me to lead the COVID-19 response at my hospital. My biggest professional obstacle to date was managing COVID-19 vaccination among healthcare workers. In Mexico, the military and central government control vaccine allocation and policy, with little room to deviate from their decisions. Navigating this bureaucracy while exercising my expertise was difficult, but I’m proud of my contributions and the outcome.

## What are the biggest drivers of your success?

Hard work, perseverance, professional integrity, and being surrounded by good people are essential to my success. Mentorship and family support are vital. I was fortunate to work with many women colleagues who, like my mother, supported and guided me through various stages of life and encouraged me not to give up.

Teaching is also a big driver of my success. Being surrounded by young learners is undoubtedly one of the most satisfying aspects of my career. Over the years, several outstanding students have become friends and colleagues, and we have achieved much together.

## Describe a pivotal mentor relationship that altered the trajectory of your career

My mentor “hall of fame” includes individuals from all stages of my career, several of whom are women. In medical school, Dr. Cecilia Ridaura, my pathophysiology teacher, became a role model as a physician and educator. Later, Dr. Linda Ward and Dr. Larry Solberg at the Mayo Clinic taught me about being an empathetic and knowledgeable clinician as well as a good scientist. The introduction to epidemiology as a career path by Dr. Juan Calva and the guidance of Dr. Patricia Volkow had a significant influence on me; their mentorship probably shaped my career the most.

Dr. Richard Wenzel was a career sponsor and memorable role model for me. We’ve often talked about our shared interests, families, and trips. Knowing him and reading about his many accomplishments is inspirational to me. His famous textbook *Prevention and Control of Nosocomial Infections* has been an essential and often-cited reference throughout my career.

I’ve been fortunate to develop mentor relationships while attending international meetings. I was thrilled to meet Dr. Trish Perl, who was chair of the session in which I was presenting an oral abstract; her advice pertaining to my research meant a lot to me. I also learned a lot from Dr. Elaine Larson on mentoring students and writing and publishing research. I am grateful for her kindness and interest in my work and professional development. She was a visionary editor of the *American Journal of Infection Control*, publishing research conducted by authors from low- and middle-income countries.

Finally, my work as SHEA International Ambassador has helped to shape my career through networking and collaboration opportunities.

## What career advice would you share with young professionals in public health, epidemiology, or stewardship?

I advise all young professionals that hard work, diligence, and perseverance are keys to success. As the pandemic has taught us, be prepared for the unexpected and keep your eyes open. You will face disappointments and successes. Dare to be different and curious, and continually approach challenges and solutions collaboratively with your colleagues and mentors. Also, surround yourself with individuals who share your interests and values, and stay in close touch with those who can help you achieve your goals and dreams. You don’t have to be an expert in methodology and statistics, but do learn the basics and collaborate with a statistician. Learn about implementation science and artificial intelligence, which will likely shape the course of medicine over the next decade.

Keep an open mind and accept new and complex challenges. Become an educator and a mentor. Remember to cherish your time outside work since a good work–life balance is sometimes hard to find.

## What books or essays most influenced you (inside or outside of science and medicine)

Over the years, several books have influenced me for different reasons. As a teenager, I was inspired by the biography of Madame Curie and Santiago Ramon y Cajal’s *Advice for a Young Investigator*.

Outside medicine, the book *Why I am not a Christian* by Bertrand Russell contains powerful messages on knowledge, kindliness, and courage. I also enjoyed *Crime and Punishment* by Fyodor Dostoevsky due to the complexity of the characters’ portrayal of human existence. The complexity and diversity of Isabel Allende’s female characters have always fascinated me. More recently, I’ve been deeply touched by *Dime Quien soy* (Tell Me Who I Am) by the Spanish-language writer Julia Navarro. The story and characters remind me who I am and where I come from.

Lately, I have been reading essays and books written by individuals leading through the COVID-19 crisis. Although it is difficult to relive certain moments, I draw satisfaction from shared experiences and lessons learned.
